# Mitochondrial Dysfunction and Diabetic Nephropathy: Nontraditional Therapeutic Opportunities

**DOI:** 10.1155/2021/1010268

**Published:** 2021-12-09

**Authors:** Ping Na Zhang, Meng Qi Zhou, Jing Guo, Hui Juan Zheng, Jingyi Tang, Chao Zhang, Yu Ning Liu, Wei Jing Liu, Yao Xian Wang

**Affiliations:** ^1^Renal Research Institution of Beijing University of Chinese Medicine and Key Laboratory of Chinese Internal Medicine of Ministry of Education and Beijing, Dongzhimen Hospital Affiliated to Beijing University of Chinese Medicine, Shipping Warehouse No. 5, Beijing 100700, China; ^2^Institute of Nephrology and Zhanjiang Key Laboratory of Prevention and Management of Chronic Kidney Disease, Guangdong Medical University, Zhanjiang, China

## Abstract

Diabetic nephropathy (DN) is a progressive microvascular diabetic complication. Growing evidence shows that persistent mitochondrial dysfunction contributes to the progression of renal diseases, including DN, as it alters mitochondrial homeostasis and, in turn, affects normal kidney function. Pharmacological regulation of mitochondrial networking is a promising therapeutic strategy for preventing and restoring renal function in DN. In this review, we have surveyed recent advances in elucidating the mitochondrial networking and signaling pathways in physiological and pathological contexts. Additionally, we have considered the contributions of nontraditional therapy that ameliorate mitochondrial dysfunction and discussed their molecular mechanism, highlighting the potential value of nontraditional therapies, such as herbal medicine and lifestyle interventions, in therapeutic interventions for DN. The generation of new insights using mitochondrial networking will facilitate further investigations on nontraditional therapies for DN.

## 1. Introduction

Diabetic nephropathy (DN) is a chronic disease that is caused by diabetes and is characterized by microangiopathy and alterations in kidney structure and function. It not only causes end-stage renal disease (ESRD) but also significantly increases the incidence and mortality rate of cardiovascular and cerebrovascular diseases [1]. With the rapid increase in the incidence of diabetes, the number of cases of DN worldwide has increased rapidly. In 2019, the International Diabetes Federation reported that approximately 463 million individuals were diagnosed with diabetes, and its incidence is expected to reach 700 million by 2045. In addition, approximately 30%–40% of these individuals are expected to develop DN [2]. However, current therapies delay rather than prevent the progression of ESRD, necessitating the search for new therapeutic targets to ameliorate the poor prognosis of DN. Current studies suggest that irregularities in key pathways and cellular components promote renal dysfunction and lead to DN. These include enhanced glucose metabolite flux, more glycation end (AGE) products, endoplasmic reticulum stress, mitochondrial dysfunction, abnormally active renin angiotensin system, and oxidative stress [3–6], with mitochondrial dysfunction playing a key role in the occurrence and pathogenesis of DN [7]. Various studies have emphasized the impact of nontraditional treatments, such as herbal medicine, nutrition, exercise, and surgical treatment, on the prevention and delayed progression of DN. Nontraditional therapy is considered a well-proven strategy which robustly improves health in most organisms. Randomized controlled clinical trials have shown that herbal medicines are efficacious and safe [8, 9]. In terms of experimental research, studies provided evidence for the efficacy of nontraditional therapies from the perspectives of ameliorating mitochondrial dysfunction. This provides a rationale for further exploration of the effect of nontraditional approaches on DN at the molecular level. Mitochondria are important for renal cell survival, as these serve as metabolic energy producers and regulate programmed cell death. The structure and function of mitochondria are regulated by a mitochondrial quality control (MQC) system, which is a series of processes that include mitochondrial biogenesis, mitochondrial proteostasis, mitochondrial dynamics/mitophagy, and mitochondria-mediated cell death. In this review, we have outlined the physiological role of mitochondria in renal function, discussed the role of mitochondrial dysfunction in the occurrence and development of DN, and emphasized on the therapeutic effect of nontraditional treatments, particularly herbal medicine (Table 1) and lifestyle interventions, on DN by targeting mitochondrial networking.

## 2. Critical Mediator of DN: Mitochondrial Dysfunction

**Figure 1:**
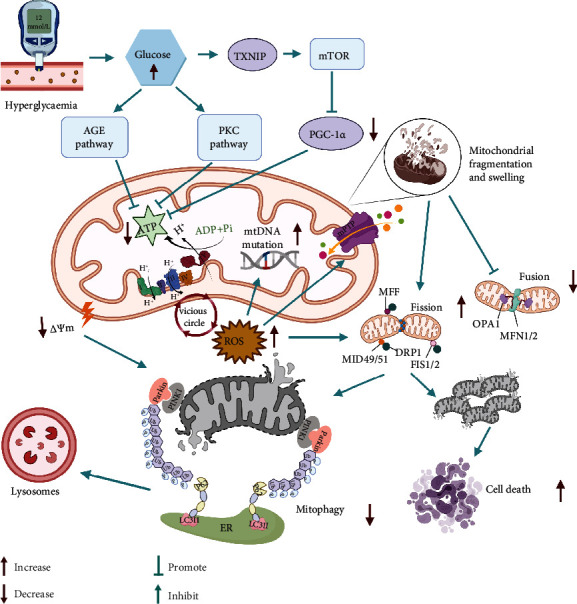
Hyperglycemia serves as the primary factor that influences mitochondrial dysfunction in DN. The increased level of glucose enhances glycolysis, and the subsequent activation of the TXNIP, AGE, and PKC pathways reinforces the decrease in ATP levels. Insufficient ATP levels stimulate the ETC to overwork in response to the energy supply for the kidneys. In turn, excessive ROS production occurs following the overactivation of the ETC, which results in decreased ATP production, mutation of mtDNA, abnormal opening of the mitochondrial permeability transition pore, and ultimately mitochondrial fragmentation and swelling. Decreases in the levels of OPA1, MFN1, and MFN2 may contribute to the decrease in mitochondrial fusion observed in DN. Activation of DRP1 promotes mitochondrial fragmentation and fission. Damaged mitochondria are cleared by mitophagy. However, an excess number of damaged mitochondria that is higher than the rate of mitophagy may result in cell death. Abbreviations: DN: diabetic nephropathy; DRP1: dynamin 1-like protein; PGC-1*α*: PGC1*α*, peroxisome proliferator-activated receptor *γ* coactivator 1*α*; AMPK: 5′-AMP-activated protein kinase; SIRT1: sirtuin-1; PINK1: putative kinase protein 1; Cyt c: cytochrome c; ROS: reactive oxygen species; MFN1 and 2: mitofusin proteins 1 and 2; OPA1: optic atrophy protein 1; MFF: mitofission proteins; FIS1: mitochondrial fission 1; PPAR: peroxisome proliferator-activated receptor; Parkin: E3 ubiquitin-protein ligase parkin; ER: endoplasmic reticulum; TXNIP: thioredoxin-interacting protein; AGE: advanced glycation end; PKC: protein kinase C; ETC: electron transport chain.

The kidney, a highly metabolic organ rich in mitochondria, requires a large amount of ATP for its normal function [30]. The kidney possesses the second highest oxygen consumption and mitochondrial content following the heart [30, 31]. Mitochondrial energetics are altered in DN due to hyperglycemia, which induces changes in the electron transport chain (ETC) which cause an increase in reactive oxygen species (ROS) and a decrease in ATP production. This leads to increased mitochondrial division, decreased PGC1*α* levels, changes in mitochondrial morphology, increased cell apoptosis, and further aggravation of the condition [32–34] (Figure 1).

### 2.1. Mitochondria: The “Energy Station” for the Kidney

In general, the mechanism of ATP production in kidney cells is determined by the cell type. For example, proximal tubules in the renal cortex are dependent on oxidative phosphorylation for ATP production to fuel active glucose, nutrient, and ion transport [35]. However, glomerular cells such as podocytes and mesangial cells are largely utilized for filtering blood, removal of small molecules (e.g., glucose, urea, salt, and water), and retaining large proteins, including hemoglobin [36]. This passive process does not require direct ATP. Therefore, glomerular cells can perform aerobic and anaerobic respiration to produce ATP for basic cellular processes [37–40]. ATP is produced through the respiratory chain, which includes five multienzyme protein complexes embedded in the inner mitochondrial membrane [19], including complex I: NADH CoQ reductase, complex II: succinate-CoQ reductase, complex III: reduced CoQ-cytochrome c reductase, complex IV: cytochrome c oxidase, and complex V: ATP synthase. One palmitate molecule produces 106 ATP molecules, whereas glucose oxidation yields only 36 ATP molecules [41, 42]. Due to the higher energy requirements of the proximal tubules, they use nonesterified fatty acids, such as palmitate, to maximize the production of ATP through *β*-oxidation. In the diabetic state, there is a large amount of substrate in the form of glucose, which provides fuel for the citric acid cycle and produces more NADH and FADH2. However, during the electron transfer process, the generation of a greater reducing force leads to electron leak; these electrons combine with oxygen molecules to produce a large amount of ROS and induce oxidative stress [43, 44].

### 2.2. ROS and Mitochondrial Dysfunction in DN: Dangerous Liaisons

The double membrane structure of mitochondria contains a large number of unsaturated fatty acids which are highly vulnerable to ROS attack. Excessive ROS results in membrane lipid peroxidation as well as triggers the mitochondrial permeability transition pore (mPTP) to abnormally open, which in turn increases its permeability and allows proteins to enter the membrane space. These negatively charged proteins are released into the cytoplasm, causing positive ions in the membrane gap to flow back into the matrix. Subsequently, the ion concentration gradient on both sides of the mitochondrial inner membrane disappears [45], mitochondrial membrane potential decreases, oxidative phosphorylation uncouples, and ATP synthesis is blocked. At the same time, it causes an imbalance of related molecules moving in and out of the mitochondria, leading to the dysfunction of the mitochondrial and cytoplasmic barriers. The greater concentration of positive ions in the mitochondrial matrix than in the cytoplasm aggravates swelling and even ruptures the mitochondria [46]. Since mitochondrial DNA (mtDNA) lacks the protection of introns, histones, and other DNA-related proteins and it is near the electron transport chain where ROS production occurs, it is more susceptible to ROS attack than nuclear DNA. Mutations may occur that lead to mitochondrial dysfunction and contribute to the progression of DN [4, 45, 47]. According to a previous study, mtDNA damage precedes bioenergy dysfunction in DN, indicating that systemic mitochondrial dysfunction and glucose-induced mtDNA changes can lead to DN [48]. In general, ROS and mitochondrial dysfunction are mutually causes and effects, forming a vicious cycle.

### 2.3. Imbalance of Mitochondrial Dynamics in DN: A Vicious Cycle

Mitochondria are highly dynamic organelles that regulate their shape, quantity, distribution, and function through continuous fusion and fission. They form a network-like mode of action in the cell which can be redistributed to meet the energy needs of the cell to the maximum extent as it is important to maintain cell homeostasis [49, 50]. Mitochondrial fusion is mainly involved in the synthesis and repair of mitochondria. When the mitochondria are slightly damaged by harmful stress, such as mtDNA variation and mitochondrial membrane potential decline, the fusion of damaged mitochondria and healthy mitochondria can repair the mutated mtDNA and restore the membrane potential to realize self-repair [51]. Mitochondrial fission also contributes to the maintenance of mitochondrial membrane potential and mtDNA stability. Depolarized mitochondrial membranes and altered mtDNA accumulate during mitochondrial fission and are discarded by autophagy or the ubiquitin-proteasome system in order to maintain normal mitochondrial function [52–54]. Increases in the levels of proteins that facilitate mitochondrial fusion occur early in the disease process in the kidneys of patients with diabetes [33]. These increases may be an early compensatory event for increased ATP demand because increasing mitochondrial fusion induced by high glucose 1 [55] or mitofusins (MFN1 and MFN2) can increase mitochondrial bioenergy function and reduce diabetic kidney damage. Fusion may also prevent renal damage in diabetes by balancing mitochondrial fission and fragmentation, which is generally considered harmful in DN.

When the mitochondrial membrane potential is damaged, the pathway for PTEN-induced putative kinase protein 1 (PINK1) to enter the inner membrane of mitochondria is blocked; therefore, it accumulates in the outer membrane, recruits Parkin to the damaged mitochondria, and phosphorylates it. Activated Parkin can ubiquitinate voltage-dependent anion channel protein 1, MFN1, MFN2, and other substrates embedded in the outer membrane. This leads to further regulation of mitochondrial morphology and dynamic changes in fission and fusion. Subsequently, the ubiquitinated mitochondria, with the assistance of autophagy receptor regulatory proteins, such as P62/SQSTM1 and microtubule-associated protein light chain 3, aggregate into double-layer autophagic vesicles, which are encapsulated to form mitochondrial autophagosomes, and fuse with lysosomes to form mitochondrial autophagic lysosomes that are degraded by hydrolases [56, 57]. Nevertheless, accumulation of autophagosomes containing mitochondria has been found in the kidneys of patients with diabetes [58] and rodent models of DN [58–60]. Although dysfunctional mitochondria can be removed by mitophagy, these can also trigger cell death in the presence of an extremely high number of damaged mitochondria relative to the rate of mitophagy [34]. Programmed cell death may occur in several forms, which include apoptosis, programmed necrosis, and autophagic cell death. Despite these distinct cell death pathways, members of the Bcl-2 family have been implicated in the direct or indirect control of mitochondrial processes [61, 62]. The permeability of the damaged mitochondrial membrane changes, resulting in the disappearance of the membrane potential, the rupture of mitochondria, and the release of intermembrane space cell death proteins (such as Cyt c, Smac/DIABLO, and HtrA2/Omi) into the cytoplasm, ultimately leading to cell death [63–65].

## 3. Maintaining Mitochondrial Homeostasis: The Target of Herbal Medicine in DN

**Figure 2:**
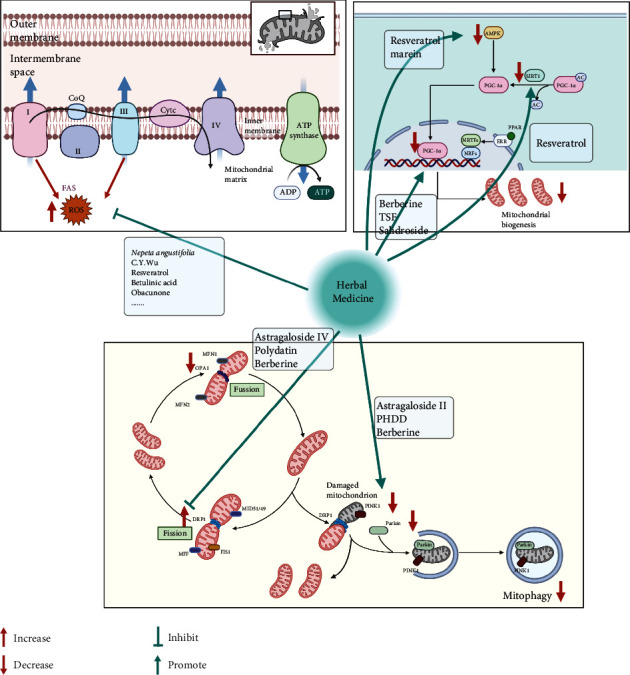
Therapeutic target of herbal medicine on mitochondrial dysfunction in DN. Herbal medicine plays a protective role in inhibiting DRP1-mediated mitochondrial dynamics to improve mitochondrial dysfunction in DN. In addition, herbal medicine enhances mitochondrial biogenesis by inducing the expression of PGC-1*α* and its upstream regulators (AMPK and SIRT1) and drives PINK1/Parkin-mediated mitophagy. In addition, the renoprotective effects of herbal medicine are associated with antioxidative stress. Abbreviations: DN: diabetic nephropathy; DRP1: dynamin 1-like protein; PGC-1*α*: PGC1*α*, peroxisome proliferator-activated receptor *γ* coactivator 1*α*; AMPK: 5′-AMP-activated protein kinase; SIRT1: sirtuin-1; PINK1: putative kinase protein 1; Cyt c: cytochrome c; ROS: reactive oxygen species; MFN1 and 2: mitofusin proteins 1 and 2; OPA1: optic atrophy protein 1; MFF: mitofission proteins; FIS1: mitochondrial fission 1; PPAR: peroxisome proliferator-activated receptor; Parkin: E3 ubiquitin-protein ligase parkin.

Mitochondrial homeostasis pertains to the balance between mitochondrial fission, fusion, and biogenesis and mitophagy, which maintains mitochondrial energetics. Diseases such as DN can disrupt mitochondrial homeostasis and thus contributes to disease progression. In recent years, most of the studies on the mechanisms of herbal medicine treatment of DN focus on improving mitochondrial homeostasis and function, aiming to restore renal function and slow the progression of DN (Figure 2).

### 3.1. Mechanism of Herb Medicine on Mitochondrial Biogenesis in DN

The complex process of mitochondrial biogenesis involves the generation of new mitochondrial mass and mtDNA replication, which are derived from preexisting mitochondria. This increases ATP production to meet the growing energy demands of cells. Mitochondrial biosynthesis is controlled by various transcriptional coactivating and coinhibitory factors [66, 67]; however, the peroxisome proliferator-activated receptor *γ* coactivator- (PGC-) 1*α* remains as the predominant upstream transcriptional regulator of mitochondrial biogenesis [68]. In several gain- and loss-of-function experimental studies, the activation of PGC-1 has been demonstrated to upregulate the expression of mitochondrial genes, including nuclear respiratory factor- (NRF-) 1, NRF-2, peroxisome proliferator-activated receptors (PPARs), and estrogen-related receptor alpha [69–71]. PGC-1*α* binds to PPARs, which act as master regulators of fatty acid oxidation (FAO) and nutrient supply [72]. To note, kidney proximal tubules have high levels of baseline energy consumption, supporting FAO as the preferred energy source in proximal tubules [73]. Defective FAO causes lipid accumulation, apoptosis, and tubule epithelial cell dedifferentiation [74]. Taken together, PGC-1*α* regulates complex processes of nutrient availability, FAO, and mitochondria biogenesis.

However, reduced PGC-1*α* expression and consequent dysfunctional mitochondria have been observed in patients with DN and animal models [6, 75–77]. Moreover, cholesterol accumulation in the kidney is a risk factor for DN progression. PGC-1*α* acts as a master regulator of lipid metabolism by regulating mitochondria [78]. Given the pivotal role of PGC-1*α* and metabolism in kidney cells, it is important to search for new approaches to restore the activity of PGC-1*α* in DN. An increasing number of studies have demonstrated that the interventional mechanisms of herbal medicines on DN are associated with this target. Berberine (BBR), an isoquinoline alkaloid present in Chinese herbal medicine (CHM), is widely used for treating DN. In particular, BBR can directly regulate PGC-1*α* to enhance FAO in DN, which promotes mitochondrial energy homeostasis and energy metabolism in podocytes [10]. Tangshen formula is a CHM that ameliorates kidney injuries in a diabetic model by promoting the PGC-1*α*-LXR-ABCA1 pathway to improve renal cholesterol efflux in db/db mice [11]. Moreover, an active component of the traditional Chinese medicine herb Rhodiola rosea L., salidroside, has been reported to greatly attenuate DN by upregulating mtDNA copy number and ETC protein expression [12].

As PGC-1*α* is almost ubiquitously expressed, targeting its upstream regulatory sensors such as 5′-AMP-activated protein kinase (AMPK), NAD-dependent protein deacetylase sirtuin-1 (SIRT1) is generally acknowledged as a significant method to restore mitochondrial function. AMPK, an extensively studied upstream regulator of PGC-1*α*, increases the rate of mitochondrial biogenesis by initiating the transcription of the *PPARGC1A* gene and by phosphorylating amino acids threonine-177 and serine-538, which in turn activates PGC-1*α*. In dead, herbal medicine prevents DN via the AMPK-SIRT1-PGC-1*α* axis that is a hot spot. Resveratrol is a naturally occurring polyphenol that imparts anti-inflammatory, antidiabetic, antioxidative, and neuroprotective effects. Particularly, resveratrol prevents DN via activation of the AMPK-SIRT1-PGC-1*α* axis, and PPARs were coactivated by PGC-1*α* in db/db mice [14]. Additional studies revealed resveratrol imparts a protective effect against DN by improving lipotoxicity, oxidative stress, and apoptosis by directly activating AdipoR1 and AdipoR2 that in turn upregulates AMPK and forkhead box protein O (FoxO) expression [13]. Interestingly, Zhang et al. further investigate the renoprotection mechanism of resveratrol *in vivo* and *in vitro*, which suggested that SIRT1/PGC-1*α* was upregulated, accompanied by improved mitochondrial function decreased oxidative stress and apoptosis [15]. In addition to single DN, the protective role of resveratrol has been verified in acute renal injury with DN via activating SIRT1–PGC–1*α*–hypoxia-inducible transcription factor-1*α* (HIF-1*α*) signaling pathways [16]. Because the role of sodium glucose cotransporter 2 (SGLT2) in excess glucose reabsorption has become a research topic of interest, SGLT2 inhibitors (SGLT2i) could reduce hyperfiltration and inhibit inflammatory and fibrotic responses that are elicited by proximal tubular cells [34, 79]. In addition, SGLT2i that enhances the excretion of urinary glucose triggers AMPK, a nutrient sensor, which in turn reverses the metabolic disorders associated with DN [80]. Marein is one of the main active components of Coreopsis tinctoria Nutt, which possesses renoprotective activity in DN by directly suppressing SGLT2 expression and then activating the AMPK–acetyl CoA carboxylase (ACC)–PGC-1*α* pathway to suppress fibrosis and inflammation [17].

### 3.2. Mechanism of Herb Medicine on Mitochondrial Dynamics in DN

Mitochondria are highly dynamic organelles that require correct mitochondrial morphology to maintain maximal ATP production [81]. The major processes of mitophagy, fission, and fusion occur as a response to mitochondrial dynamics as well as to maintain mitochondrial integrity in different metabolic conditions. Fission is essential in the isolation of damaged parts from the rest of the mitochondrial network and is induced by translocating dynamin 1-like protein (DRP1) from the cytosol to the outer membrane of the mitochondria where it binds to its receptors, including mitochondrial fission 1 (FIS1), mitochondrial fission factor (MFF), and mitochondrial dynamics proteins MID49 and MID51 [82, 83]. Mitochondria fusion involves the recruitment of a series of proteins that include MFN1 and MFN2 that triggers outer membrane fusion, as well as optic atrophy protein 1 (OPA1) that facilitates inner membrane fusion. However, in DN, excessive mitochondrial fission and fusion are associated with key features of renal damage. Mitochondrial dysfunction in podocytes is increasingly recognized as a factor contributing to the pathogenesis of DN [84].

Previous studies have elucidated the correlation between mitochondrial dynamics disorder and DN progression and revealed that DRP1 may be potentially utilized as a therapeutic target in the treatment of DN [85]. Meanwhile, traditional Chinese medicine has definite effects on this point. Besides its function on PGC-1*α*-mediated mitochondrial biogenesis, BBR plays a therapeutic role in positively regulating DRP1-mediated mitochondrial dynamics to protect glomerulus and improve the fragmentation and dysfunction of mitochondria in podocytes [18]. AS-IV is a major and active component of *Astragalus*, which is a traditional Chinese medicinal herb for tonifying. Liu et al. have shown in diabetic db/db mice that AS-IV significantly improves albuminuria and renal pathologic injury. In addition, they found AS-IV decreased the elevation of renal DRP1, Fis1, and MFF expression in db/db mice [19]. More than that, polydatin which is mainly extracted from the roots of Polygonum cuspidatum not only inhibits DRP1 activation and fragmented mitochondria caused by high glucose but also blocked the increase of apoptosis through a DRP1-dependent mechanism [20, 86].

Mitophagy allows the removal of damaged and nonfunctional mitochondria from the network and requires the efficient recognition of targeted mitochondria followed by the engulfment of mitochondria by autophagosomes [87]. As part of a healthy network of mitochondria, mitophagy is regulated by a PINK1-PARKIN pathway for mitochondrial identification and labeling [88]. However, impairment of the mitophagy system aggravated the progression of DN, which was mainly caused by decreases in renal PINK1 and Parkin expression in diabetes following activation of either FOXO1 or NRF2 signal [89, 90]. In recent years, the role and regulation of mitophagy in DN have attracted lots of attention. It has been reported that AS II, another of the active constituents of Astragalus, exerts protective effects on podocyte injury and mitochondrial dysfunction through enhancing mitophagy activation via modulation of NRF2 and PINK1 [21]. Huangqi-Danshen decoction which mainly includes Astragali Radix (Huang-qi) and Salviae Miltiorrhizae Radix et Rhizoma (Dan-shen) significantly alleviated DN, which might be associated with the reversion of the enhanced mitochondrial fission and the inhibition of PINK1/Parkin-mediated mitophagy [22].

## 4. The Modulation of Mitochondrial ROS: The Effect of Herb Medicine in DN

Increased oxidative stress is due to ROS production caused by dysfunctional cellular respiration during hyperglycemia and is the major pathway involved in the pathogenesis of DN [3]. In the early phase of pathogenesis, ROS mainly from mitochondria origin do have a role in the regulation of various metabolic pathways. However, their accumulation that exceeds local antioxidant capacity is a biomarker of mitochondrial dysfunction in DN [91]. Overproduction of ROS can subsequently induce oxidative stress and cause damage to critical cellular components (particularly protein and DNA) and glomerular podocyte, which contributed to inflammation, interstitial fibrosis, and apoptosis [92, 93]. The damaging effect of ROS is thought to be mediated by activation of several pathways such as NF-*κ*B, hexosamine, and the formation of AGE products [94]. The transcription of genes that encode antioxidant enzymes, including SOD2, glutathione peroxidase, and catalase, is activated by NRF2, which in turn promotes antioxidant activity and induces negative feedback on NF-*κ*B [95, 96]. Therefore, an emerging therapeutic target antioxidant defense mechanism and promoting renoprotection in DN involves the activation of NRF2 with its mediated antioxidant enzymes [97], while traditional Chinese medicine has definite antioxidant effects.

*Nepeta angustifolia* C. Y. Wu, an important medicinal material constituting a variety of traditional Chinese medicine prescription, has significant antioxidant activity [98]. *Nepeta angustifolia* C. Y. Wu inhibits proinflammatory mediators and renal oxidative stress in diabetic rats, as well as improves mitochondrial potential to disrupt mesangial cell apoptosis caused by oxidative stress *in vitro* [23]. AMPK is an energy sensor in metabolic homeostasis [99]. Recent studies have shown that AMPK participates in the attenuation of oxidative stress in DN [100]. The beneficial effects of resveratrol on renal diseases are attributed to its antioxidative properties. Moreover, the study conducted by Kitada et al. [24] indicated that resveratrol can enhance mitochondrial biogenesis and protect against DN through normalisation of Mn-SOD dysfunction via the AMPK/SIRT1-independent pathway. Betulinic acid is extracted from the outer bark of white birch trees and exerts a protective effect on DN by effectively attenuating oxidative stress and inflammatory conditions via the AMPK/NF-*κ*B/NRF2 signaling pathway [25]. Zhou et al. showed that obacunone, a natural bioactive compound isolated from the Rutaceae family, blocks GSK-3*β* signal transduction and subsequently enhances the activity of NRF2 to inhibit oxidative stress and mitochondrial dysfunction in NRK-52E cells [26]. Furthermore, Yahya et al. [27] showed using STZ-induced diabetic rats that curcumin imparts a nephroprotective effect via NRF2 activation, inhibition of NF-*κ*B, suppression of NADPH oxidase, and downregulation/inhibition the PKC *β*II/p66 Shc axis. The role of herbal medicine in NRF2/AGE signal should also be carefully considered as NRF2/AGE plays a pivotal role in controlling transcriptional regulation of the genes encoding endogenous antioxidant enzymes [97]. Notoginsenoside R1, a novel phytoestrogen isolated from *Panax notoginseng* (Burk.) F. H. Chen, was found to decrease AGE-induced mitochondrial injury and promote NRF2 and HO-1 expression to eliminate oxidative stress and apoptosis in DN [28]. Further, oleanolic acid combined with N-acetylcysteine has therapeutic effects on DN through an antioxidative effect and endoplasmic reticulum stress reduction by the NRF2/Keap1 system [29].

## 5. Lifestyle Interventions

Diabetes is usually accompanied by excessive nutrition and calories, as well as a decrease in physical activity, both of which aggravate nephropathy [91]. In 2020, the consensus statement of the American Association of Clinical Endocrinologists and American College of Endocrinology on the comprehensive management algorithm for type 2 diabetes (T2D) mentioned that lifestyle optimization is essential for all diabetic patients, including healthy eating patterns, weight loss, physical activity, and smoking cessation [101]. We summarize therapeutic strategies about lifestyle intervention, with a focus on mitochondrial biogenesis, to improve the malignant progress of DN.

### 5.1. Healthy Eating Patterns and DN

Healthy eating patterns are important for patients with diabetes and DN to maintain glucose control and inhibit the progression of kidney damage [102]. Particularly in late-stage kidney disease, a low-protein diet (LPD) can maintain the renal function in patients with chronic kidney disease (CKD), including those with DN [103–106]. In terms of the molecular mechanism of LPD against DN, earlier animal studies have revealed that LPD decreases intraglomerular pressure via reduction of afferent arteriole vasoconstriction, which in turn improves glomerular hyperfiltration and hypertension as well as reduces fibrosis of mesangial cells via growth factor-*β* signals. Furthermore, an LPD, particularly a very LPD, can also prevent renal tubular cell injury, apoptosis, inflammation/oxidative stress, and fibrosis within the tubule-interstitial region by reducing the accumulation damaged mitochondria, which is triggered by the reduction in the activity of mammalian target of rapamycin complex 1 and the restoration of autophagy [107]. However, due to the insufficiency of clear results from present clinical trials, the renal protective effect of LPD against DN is controversial. Existing clinical research evidence is unable to fully prove the renal protective effect of LPD [108–110], although other studies have shown that LPD can delay the decline of renal function [111, 112]. In addition, the American Diabetes Association believes that a short-term (approximately 3–4 months) low-carbohydrate (LC) diet is beneficial for diabetes management [113]. Compared with an ordinary diet, a LC diet contains a higher protein and fat content and ratio. The energy required by the body mainly comes from the metabolism of fat into ketone bodies; therefore, it is also called a ketogenic diet. A LC diet can directly reduce blood sugar levels, and ketone bodies have various functions, such as anti-inflammatory, mitochondrial biogenesis regulatory, and antioxidant activity [114]. However, a long-term LC diet may damage kidney function, which is mainly attributed to its high protein content [113]. Several human physiological studies have shown that a high-protein diet can cause renal hyperfiltration [115–117]. Although the actual cause of this phenomenon remains unclear, studies have attempted to describe the effects related to specific amino acid components as well as dietary advanced glycosylation end products [118, 119].

### 5.2. Weight Loss and DN

In a review on weight loss in coronary heart disease, the GFR and proteinuria in patients with weight loss improved, and the weight loss and CKD index effects of surgical intervention were better than those of drug and lifestyle interventions [120–122]. Miras et al. [123] confirmed this finding by retrospectively analysing data of 84 patients with DN who underwent bariatric surgery over a 12–18-month period. Among them, 32 patients with albuminuria at baseline had a mean 3.5-fold decrease in the postoperative albumin–creatinine ratio, and albuminuria in 32 patients returned to normal levels.

A systematic review and meta-analysis including approximately 30 studies reported the impact of bariatric surgery on renal function. All studies measured the changes in relevant indicators of renal dysfunction within 4 weeks before and after bariatric surgery. Among them, six studies measured a 54% reduction in the risk of postoperative glomerular hyperfiltration, and 16 studies measured a 60%–70% reduction in the risk of postoperative albuminuria and total proteinuria [124]. Cohort studies have reported the benefits of bariatric surgery in improving creatinine levels and the GFR or reducing the incidence of stage 4 ESRD [124–128], which may be related to improved renal tubular damage [129]. Furthermore, surgery-induced weight loss can improve mitochondrial biogenesis and mitochondrial dysfunction [70], which may be an effective treatment for DN [130, 131].

### 5.3. Physical Activity and DN

Moderate aerobic exercise can reduce weight and improve insulin sensitivity, hyperglycemia, hyperlipidemia, and DN [132, 133]. Studies have reported that upregulation of the expression of eNOS and nNOS proteins in the kidney and improvement in NADPH oxidase and *α*-oxyaldehyde levels may reduce early diabetic nephropathy in Zucker diabetic fatty rats. Chronic aerobic exercise has health benefits and may be utilized as a treatment method for the prevention and development of renal dysfunction in T2D [134]. However, strenuous exercise may aggravate DN progression. Studies have reported that the rates of urinary protein excretion increase after strenuous exercise and tubular proteinuria occurs [107, 135]. A prospective study has demonstrated for the first time that the intensity of physical activity, rather than the total amount, is associated with the occurrence and progression of DN in type 1 diabetes. Moreover, the beneficial relationship between moderate- and high-intensity physical activity and progression of nephropathy is not affected by the duration of diabetes, age, sex, or smoking [136].

This high-intensity, low-volume training program not only increases the content of citrate synthase and cytochrome C oxidase subunit IV with increasing insulin sensitivity but also stimulates mitochondrial biogenesis. The contraction activity can lead to important signal events such as calcium release, AMP/ATP ratio change, cell redox state, and ROS generation. These events activate AMPK and stimulate PGC-1*α* [137]. PGC-1*α* can stimulate several genes encoding mitochondrial proteins, mtDNA amplification and proliferation, and oxidative metabolism. In short, the number of mitochondria per cell and their function increased several times in trained subjects compared to those in sedentary subjects. Although the best exercise type, frequency, and intensity for preventing DN or DN progression have not been formally determined, it is recommended to perform moderate-to-high-intensity aerobic exercise for at least 150 minutes and two to three sessions of resistance exercise per week for patients without contraindications [138].

## 6. Conclusion

Recently, mitochondrial dysfunction has been shown to be a critical determinant of the progressive loss of renal function in patients with diabetes. Pharmacological regulation of mitochondrial networking may be a promising therapeutic strategy in preventing and treating DN. Moreover, nontraditional therapies, including herbal medicine and lifestyle interventions, play a renoprotective role in improving mitochondrial homeostasis and function. Overall, the interventional mechanisms of nontraditional therapies for DN are still in their infancy compared with traditional treatments. Elucidating the mechanism of action and efficacy of nontraditional therapies involving mitochondria may facilitate the discovery of novel therapeutic approaches in treating DN and preventing the progression of DN to ESRD.

## Figures and Tables

**Table 1:** Mitochondria-targeted herb medicine in DN.

Herb medicine	The form of herb medicine	Experimental model	Target	Pathway	Observed effect	Ref.
			*Mitochondrial biogenesis*			
Berberine	Pure chemical	Patients with DN, db/db diabetic mice	PCG-1*α*↑, FAO↑, AMPK↑	PGC-1*α* signaling pathway	Restoration of PGC-1*α* activity and the energy homeostasis	[10]
Tangshen formula	Extract	db/db diabetic mice, mTECs	PGC-1*α*↑, LXR↑, ABCA1↑	PGC-1*α*-LXR-ABCA1 pathway	Improving cholesterol efflux	[11]
Salidroside	Pure chemical	db/db diabetic mice	SIRT1↑, PGC-1*α*↑	SIRT1/PGC-1*α* axis	Improving mitochondrial biogenesis	[12]
Resveratrol	Pure chemical	db/db diabetic mice, HGECs	AdipoR1↑, AdipoR2↑, AMPK↑, SIRT1↑, PGC-1*α*↑, PPAR*α*↑	AMPK–SIRT1–PGC–1*α* axis	Ameliorating lipotoxicity, oxidative stress, apoptosis, and endothelial dysfunction	[13]
Resveratrol	Pure chemical	db/db diabetic mice	AMPK↑, SIRT1↑, PGC-1*α*↑, PPAR*α*↑	AMPK–SIRT1–PGC–1*α* axis	Prevention of lipotoxicity-related apoptosis and oxidative stress	[14]
Resveratrol	Pure chemical	STZ-induced diabetic rats, podocytes	SIRT1↑, PGC-1*α*↑, ROS↓	SIRT1/PGC-1*α* axis	Inhibition of mitochondrial oxidative stress and apoptosis	[15]
Resveratrol	Pure chemical	DN rabbits with AKI, HK-2 cells	SIRT1↑, PGC-1*α*↑, HIF-1*α*↓	SIRT1–PGC–1*α*–HIF-1*α* signaling pathways	Reducing renal hypoxia, mitochondrial dysfunction and renal tubular cell apoptosis	[16]
Marein	Extract	db/db diabetic mice, HK-2 cells	SGLT2↓, SREBP-1↓, AMPK↑, PGC-1*α*↑	AMPK/ACC/PGC-1*α* pathway	Amelioration of fibrosis and inflammation	[17]
			*Mitochondrial dynamics*			
Berberine	Pure chemical	db/db diabetic mice, podocytes	DRP1↓, MFF↓, FIS1↓, MID49↓, MID51↓, PGC-1*α*↑	DRP1 modulator	Inhibiting mitochondrial fission and cell apoptosis	[18]
Astragaloside IV	Pure chemical	db/db diabetic mice	Drp1↓, MFF↓, Fis1↓	Mitochondrial quality control network	Amelioration of renal injury	[19]
Polydatin	Pure chemical	KKAy mice, hyperglycemia-induced MPC5 cells	DRP1↓, ROS↓, caspase-3↓, caspase-9↓	ROS/DRP1/mitochondrial fission/apoptosis pathway	Impairing mitochondria fitness and ameliorating podocyte injury	[20]
			*Mitophagy*			
Astragaloside II	Pure chemical	STZ-induced diabetic rats	NRF2↑, Keap1↓, PINK1↑, Parkin↑	NRF2 and PINK1 pathway	Amelioration of podocyte injury and mitochondrial dysfunction	[21]
Huangqi-Danshen decoction	Extract	db/db diabetic mice	DRP-1↓, PINK1↑, Parkin↑	PINK1/Parkin pathway	Protection kidney injury by inhibiting PINK1/Parkin-mediated mitophagy	[22]
			*Mitochondria ROS*			
Nepeta angustifolia C. Y. Wu	Extract	HFD/STZ-induced diabetic rats, mesangial cells	SOD↑, ROS↓, MDA↓	Mitochondrial-caspase apoptosis pathway	Antioxidative stress, inflammation and inhibiting mesangial cell apoptosis	[23]
Resveratrol	Pure chemical	db/db diabetic mice	ROS↓, AMPK↑, SIRT1	AMPK/SIRT1-independent pathway	Antioxidative stress and enhanced mitochondrial biogenesis	[24]
Betulinic acid	Pure chemical	STZ-induced diabetic rats	SOD↑, CAT ↑, MDA↓, AMPK, NF-*κ*B↓, NRF2↑	AMPK/NF-*κ*B/NRF2 signaling pathway	Attenuating the oxidative stress and inflammatory condition	[25]
Obacunone	Pure chemical	NRK-52E cells	SOD↑, GSK-3*β*↓, NRF2↑	GSK-3*β*/Fyn pathway	Inhibiting oxidative stress and mitochondrial dysfunction	[26]
Curcumin	Pure chemical	STZ-induced diabetic rats	NRF2↑, FOXO-3a↑, PKC*β*II↓, NF-*κ*B↓	PKC *β*II/p 66 Shc axis	Antioxidative stress	[27]
Notoginsenoside R1	Pure chemical	db/db diabetic mice, HK-2 cells	ROS↓, NRF2↑, HO-1↑	NRF2 pathway	Inhibition of apoptosis and renal fibrosis caused by oxidative stress	[28]
Oleanolic acid and N-acetylcysteine	Pure chemical	Type 2 diabetic rat model, mesangial cells	ROS↓, NRF2↑, TGF-*β*/smad2/3↓, *α*-SMA↓	NRF2/Keap1 system	Inhibition of oxidative stress and ER stress	[29]
